# Electronic-Structure-Modulated Cu,Co-Coanchored N-Doped Nanocarbon as a Difunctional Electrocatalyst for Hydrogen Evolution and Oxygen Reduction Reactions

**DOI:** 10.3390/molecules29132973

**Published:** 2024-06-22

**Authors:** Liyun Cao, Rui Liu, Yixuan Huang, Dewei Chu, Mengyao Li, Guoting Xu, Xiaoyi Li, Jianfeng Huang, Yong Zhao, Liangliang Feng

**Affiliations:** 1 School of Materials Science and Engineering, International S&T Cooperation Foundation of Shaanxi Province, Shaanxi University of Science and Technology, Xi’an 710021, China; caoliyun@sust.edu.cn (L.C.); liurui202105@163.com (R.L.); lixiaoyi202104@163.com (X.L.); 220222078@sust.edu.cn (Y.Z.); 2 School of Materials Science and Engineering, The University of New South Wales, Sydney, NSW 2052, Australia; z5485592@ad.unsw.edu.au (Y.H.); d.chu@unsw.edu.au (D.C.); mengyao.li1@unsw.edu.au (M.L.); 3 College of Chemistry and Environmental Sciences, Kashi University, Kashi 844000, China; xguoting@163.com

**Keywords:** Cu-based materials, heterostructure, oxygen reduction reaction, hydrogen evolution reaction

## Abstract

To alleviate the problems of environmental pollution and energy crisis, aggressive development of clean and alternative energy technologies, in particular, water splitting, metal–air batteries, and fuel cells involving two key half reactions comprising hydrogen evolution reaction (HER) and oxygen reduction (ORR), is crucial. In this work, an innovative hybrid comprising heterogeneous Cu/Co bimetallic nanoparticles homogeneously dispersed on a nitrogen-doped carbon layer (Cu/Co/NC) was constructed as a bifunctional electrocatalyst toward HER and ORR via a hydrothermal reaction along with post-solid-phase sintering technique. Thanks to the interfacial coupling and electronic synergism between the Cu and Co bimetallic nanoparticles, the Cu/Co/NC catalyst showed improved catalytic ORR activity with a half-wave potential of 0.865 V and an excellent stability of more than 30 h, even compared to 20 wt% Pt/C. The Cu/Co/NC catalyst also exhibited excellent HER catalytic performance with an overpotential of below 149 mV at 10 mA/cm^2^ and long-term operation for over 30 h.

## 1. Introduction

The exhaustion of natural resources and ecological imbalance caused by the massive consumption of fossil energy accelerates global warming and thus has become a major and urgent problem to be solved globally [1,2,3]. The search for energy storage and conversion technologies to develop effective green renewable energy sources is an effective solution to the above problems [4,5,6]. The efficiency of all the electrochemical processes involved, such as hydrogen evolution reaction (HER) for electrochemical water splitting to produce hydrogen energy [7,8] and oxygen reduction reaction (ORR) for cathodes of fuel cells and metal-air batteries used for energy conversion [9,10], is the most significant constraint to the development of energy conversion devices.

Efficient electrocatalysts can effectively reduce energy consumption during electrocatalytic reactions, so the research of feasible and efficient electrocatalysts is one of the most important aspects in advancing sustainable energy technologies. The primary catalysts for HER and ORR are Pt-based catalysts, but they have severely limited large-scale development and application in commerce due to the expense and scarcity of Pt [11,12,13]. Hence, the development of inexpensive, efficient, durable, and green electrocatalysts has become a priority direction of current research [14,15].

Aiming at minimizing the cost, in recent years, researchers have made efforts to exploit non-precious-metal-based catalysts, such as metallic elements [16], phosphides [17], sulfides [18], and carbides [19], to eliminate platinum-based catalysts. Transition metal Cu-based materials are very abundant in the earth’s crust, inexpensive, and highly conductive have attracted the attention of researchers [20,21,22]. Theoretical calculations have reported that Cu has the highest ORR activity among all non-precious transition metals because of its position close to Pt at the top of the “volcano diagram” [23]. Nevertheless, its remarkably low intrinsic activity limits its application within the domain of electrocatalytic hydrogen evolution reaction [24].

Bimetallic catalysts, which have demonstrated better catalytic activity and stability than monometallic catalysts, have received growing attention in the field of electroanalysis over the past few decades [25]. Nunes et al. [26] reported a composite electrocatalyst composed of high-specific-surface-area graphite (HSAG)-loaded Cu-Pd nanoparticles (NPs). Compared to the loading of Cu and Pd alone, due to the coordinative effect between Cu and Pd nanoparticles, the bimetallic Cu-Pd/HSAG composites showed the most stable ORR catalytic activity in alkaline media and tolerance to methanol, and the current drop was less than 10% after the addition of methanol. Sanad et al. [27] prepared a Cu-Co MOF electrocatalyst that possessed ultra-high ORR catalytic activity that exceeds that of Pt/C for onset potential, half-wave potential, and electrochemical stability due to the Co and Cu interactions facilitating electron coupling, leading to the enhancement of the interatomic electron transfer rate. Kato et al. [28] reported ORR performed using (Cu, Fe)-N-CNT in acidic media, and exhibited high ORR catalytic activity. The analysis showed that the coexistence of Fe and Cu active sites made the 4e^−^ direct transfer pathway the only pathway, thus avoiding the production of by-product H_2_O_2_ in the ORR.

Owing to the fact that commonly used transition metals are very active at high temperatures, the pyrolysis process usually causes the exodus and aggregation of metal nanostructure, which leads to catalyst deactivation [29]. In contrast, the combination of transition metals with a carbon matrix can well alleviate the agglomeration phenomenon and improve the metal’s electrical conductivity. More notably, transition metals can easily coordinate with nitrogen to form M-N structures as highly active sites, which is favorable for HER and ORR performance [30,31,32,33,34]. Zhou et al. [35] made platinum-based nanoparticles uniformly anchored on porous carbon carriers, which could effectively avoid the dissolution and aggregation of metals and expose more active sites due to the electronic and cooperative effects between the platinum and the carriers, thus improving the performance of the composite catalyst. Therefore, the development of bifunctional Cu/Co bimetallic heterogeneous nanoparticles loaded on N-doped carbon for efficient HER and ORR is well worth exploring.

In this work, we have successfully prepared a novel composite with bifunctional electrocatalytic properties by introducing the transition metal cobalt with heterogeneous Cu/Co bimetallic nanoparticles uniformly dispersed on N-doped carbon layers (Cu/Co/NC). The prepared Cu/Co/NC composite exhibits good catalytic performance, and as expected, Cu/Co/NC shows excellent HER performance in alkaline media, requiring only 149 mV to achieve 10 mA/cm^2^ for 30 h of simultaneous stable operation. In addition, Cu/Co/NC showed excellent ORR performance with E_1/2_ = 0.865 V, good stability, and methanol resistance, even compared to 20 wt% Pt/C. The present study opens up a new pathway for the rational development of bifunctional electrocatalysts with cost-neutral and highly efficient Cu/Co bimetallic heterostructures.

## 2. Results and Discussion

### 2.1. Structure and Characterisation of Cu/Co/NC Materials

**Figure 1:**
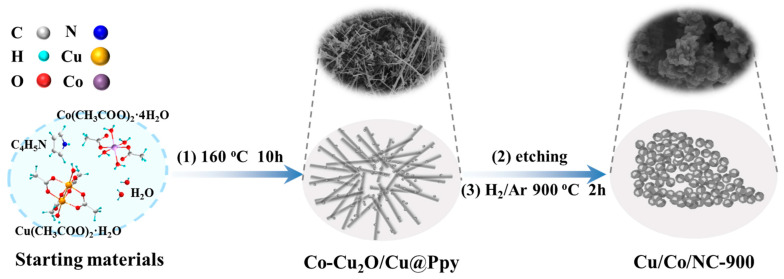
Schematic illustration of the synthesis of Cu/Co@NC-900.

The synthesis process of Cu/Co/NC materials is represented in Figure 1. Firstly, a hydrothermal reaction was performed at 160 °C for 10 h to obtain the precursor Co-Cu_2_O/Cu@Ppy (see XRD in Figure S1), in which Cu(CH_3_COO)_2_·H_2_O was utilized as the copper source, Co(CH_3_COO)_2_·4H_2_O was utilized as the cobalt source, and pyrrole monomer was utilized as the carbon source, respectively. Then, Cu and part of Cu_2_O in the precursor Co-Cu_2_O/Cu@Ppy were etched out by sodium thiosulfate solution to acquire the precursor Co-Cu_2_O@Ppy (Figure S1), which was calcined at 900 °C for 2 h under a reducing atmosphere of H_2_/Ar to finally produce the intended product Cu/Co/NC-900.

**Figure 2:**
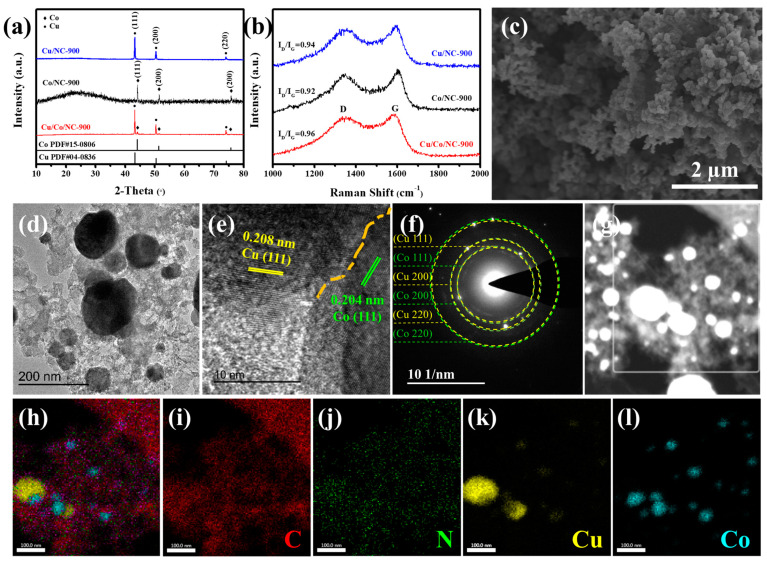
Microstructural characterization of Cu/Co/NC-900. (**a**) XRD image, (**b**) Raman image, (**c**) SEM image, (**d**) TEM image, (**e**) HRTEM image, (**f**) SAED pattern of the Cu/Co/NC-900, (**g**) STEM image (The white box is the selected area of EDX mapping images and corresponding), and (**h**–**l**) EDX mapping images of C, N, Cu, and Co elements in Cu/Co/NC-900.

As illustrated in Figure 2a, the characteristic peaks of Cu/Co/NC-900 detected at 2θ values of around 43.30°, 50.43°, and 74.13° are matched to (111), (200), and (220) crystal facets of the cubic phase Cu (PDF#04-0836). The rest of diffraction peaks located at around 44.22°, 51.52°, and 75.85°are in agreement with the (111), (200), and (220) crystal planes of the cubic Co (PDF#15-0806). This shows that Cu/Co/NC is composed of Cu and Co. To investigate the role of Cu and Co, the XRD patterns of Co/NC-900 and Cu/NC-900 are shown in Figure 2a. As shown in Figure 2b, two distinct and unique characteristic bands appear in all three samples, corresponding to the D band of sp^3^ orbital hybridization of carbon representing disordered structures and the G band of sp^2^ orbital hybridization of carbon representing graphitic structures at about 1350 cm^−1^ and 1590 cm^−1^, respectively. The magnitude of intensity ratio between D-band and G-band (I_D_/I_G_) evaluates the degree of disorganization within the material, and a higher I_D_/I_G_ value indicates that the material has more disordered structures, which means that the material has more defective structures and lower electrical conductivity. The maximum I_D_/I_G_ value of Cu/Co/NC-900 is 0.96, which is higher than that of Co/NC-900 (I_D_/I_G_ = 0.92) and Cu/NC-900 (I_D_/I_G_ = 0.94), indicating that there are a multitude of dopant sites or rich defects in the carbon layer of the Cu/Co/NC-900 samples, contributing to enhancing the electron transfer ability and the exposure of active sites [36]. The heat treatment temperature was also found to have no effect on the composition of the physical phase, as distinct peaks of copper and cobalt were detected in all samples obtained at the same treatment temperature, as shown in Figure S2a. As shown in Figure S2b, the calculated I_D_/I_G_ ratio for Cu/Co/NC-900 is 0.96, which is above the 0.92 seen with Cu/Co/NC-800 and below the 0.97 seen with Cu/Co/NC-1000. The comparison shows that Cu/Co/NC-900 possesses a better degree of disorderedness with a large variety of structural defects to expose more active sites compared to Cu/Co/NC-800, whereas it possesses better electrical conductivity than Cu/Co/NC-1000 with a better degree of graphitization. Figure S3 further illustrates that Cu/Co/NC-800 nanoparticles at a lower temperature grew unevenly (Figure S3a), and the Cu/Co/NC-1000 nanoparticles are prone to aggregation due to the high temperature, which does not favor adequate access to the electrolyte, and limits the exposure of active sites. As shown in Figure S3b, the morphology for the Cu/Co/NC-900 sample presents a well-dispersed and intact nanoparticle structure, which facilitates an adequate specific surface area of the material as well as adequate contact with the electrolyte, leading to the exposure of a multitude of active sites, which results in the enhancement of electrocatalytic activity. This shows that the morphology of nanoparticles is more homogeneous at a heat treatment temperature of 900 °C, and the temperature is one of the important factors affecting the homogeneous generation of nanoparticles.

The morphology and microstructure of Cu/Co/NC-900 are shown in Figure 2c–h and Figure S4b, respectively. The SEM image of Cu/Co/NC-900 in Figure 2c shows a uniformly distributed nanoparticle structure. Whereas, both Cu/NC-900 and Co/NC-900 (Figure S4a,c) exhibit inhomogeneous nanoparticle structures, indicating that Cu and Co interaction inhibits nanoparticle agglomeration, thereby enlarging the active surface area in the material. We used TEM to probe the microscopic morphology of the samples. Figure 2d shows that the Cu/Co/NC-900 sample exhibits nanoparticles loaded on the N-doped carbon layer and the particle size for the Cu nanoparticles is about 100 nm and that for the Co nanoparticles is about 50 nm. The HRTEM image of Figure 2e further reveals that Cu and Co nanoparticles in Cu/Co/NC-900 have a strong interwoven incoherent interface, in which the crystal facet spacing of 0.208 nm belongs to the (111) crystal facet of Cu, and the crystal facet spacing of 0.204 nm corresponds to the (111) facet of Co, consistent with the XRD (Figure 2a). In addition, the selected area electron diffraction (SAED) image of Cu/Co/NC-900 (Figure 2f) shows several bright rings attributed to the (111), (200), and (220) crystal planes of Cu and Co, elucidating that Cu/Co/NC-900 comprises a polycrystalline structure. The STEM images and the matching EDS maps (Figure 2i–l) show that the elements C, N, Cu, and Co are present and uniformly dispersed on the carbon layer. The heterogeneous interface formed between Cu and Co supplies increased interfacial area, which in turn, enhances the number of active sites on the surface, and boosts the rate of electron transfer, thereby speeding up the kinetic process of the electrocatalytic reaction.

**Figure 3:**
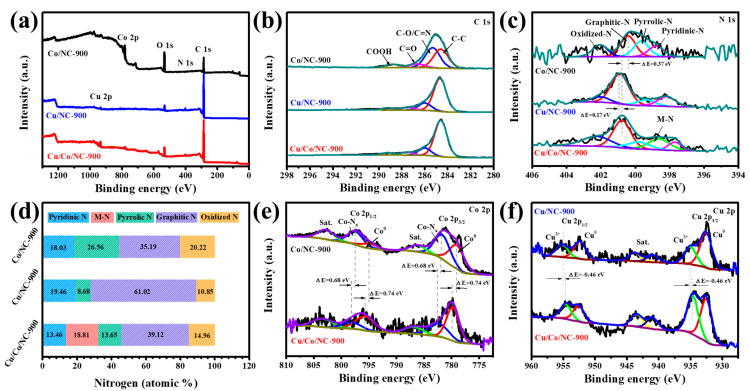
(**a**) Survey, (**b**) C 1s, (**c**) N 1s XPS spectra of Cu/Co/NC-900, Cu/NC-900 and Co/NC-900, (**d**) The contents of pyridinic N, M-N (M = Cu, Co), pyrrolic N, graphitic N, oxidized N (acquired from XPS) of three samples, (**e**) Co 2p XPS spectra of Cu/Co/NC-900, and Co/NC-900, and (**f**) Cu 2p XPS spectra of Cu/Co/NC-900, and Cu/NC-900.

XPS characterization was used to further determine the elemental composition and electron distribution. The presence of five elements (C, N, O, Co, and Cu) in the Cu/Co/NC-900 sample can be seen from the full spectrum in Figure 3a. The high-resolution C 1s XPS spectrum of the Cu/Co/NC-900 sample (Figure 3b) shows peaks at 289.26 eV, 287.53 eV, 286.00 eV, and 284.60 eV, which correspond to COOH, C=O, C-O/C=N, and C-C [37], where the presence of C=N bond indicates the successful doping of nitrogen into the carbon matrix [38]. In the Cu/Co/NC samples, five nitrogen structures were detected in the N 1s signal band (Figure 3c): pyridine nitrogen (397.72 eV), metal nitrogen bond (398.82 eV) (metal: Cu or Co), pyrrole nitrogen (400.02 eV), graphite nitrogen (400.92 eV), and nitrogen oxide (402.42 eV) [39]. It is noteworthy that the peaks of the pyridine N in the Cu/Co/NC-900 sample both shifted towards lower binding energies by 0.44 eV and 1.08 eV, respectively, compared to the pure-phase Cu/NC-900 vs. Co/NC-900. The shift in the peaks of pyridine N in the Cu/Co/NC-900 sample is attributed to the synergistic impact of the creation of the heterointerface by Cu/Co, which promotes Cu-N and Co-N bond generation, thus driving the electronic delocalization of the conjugated heptazine framework to enhance the electron separation rate [40]. Meanwhile, the XPS peak of graphitic nitrogen in the Cu/Co/NC-900 sample shifted toward a higher binding energy compared to that of Co/NC-900 and toward a lower binding energy compared to that of Cu/NC-900, which implies that the N can regulate the electron configuration of Cu/Co/NC-900 [41]. From the distribution of N content in Figure 3d, it can be seen that the content of M-N in the Cu/Co/NC-900 sample is as high as 18.81%, which has been reported to be a very efficient active site during the electrocatalytic reaction, and thus facilitates the promotion of the catalytic reaction [42]. According to Figure 3e, the peaks at 779.76 and 795.66 eV in the Co 2p spectra of Cu/Co/NC-900 are attributed to Co 2p_2/3_ and Co 2p_1/3_ of Co^0^, while the peaks at 782.31 and 798.11 eV are attributed to Co 2p_2/3_ versus Co 2p_1/3_ of Co-N_x_ [43]. Then, in the Cu 2p spectrum of Cu/Co/NC-900 (Figure 3f), the peaks centered at 932.53 and 952.55 eV are ascribed to Cu 2p_2/3_ and Cu 2p_1/3_ of Cu^0^, and the peaks centered at 934.65 and 954.62 eV are attributed to Cu 2p_2/3_ and Cu 2p_1/3_ of Cu^II^ [44]. Furthermore, the binding energies of Cu/Co/NC-900 positively shifted by 0.74 eV for Co^0^ and positively shifted by 0.68 eV for Co-N_x_, while the binding energy of Cu^II^ negatively shifted by 0.46 eV for Cu/Co/NC-900 compared to Co/NC-900. This implies that the binding energies of the metal Cu and Co nanoparticles are coupled to each other to form a heterojunction interface with strong electronic interactions, so Cu receives electrons while Co loses electrons, which facilitates charge transfer and reaction kinetics during electrocatalysis [25].

**Figure 4:**
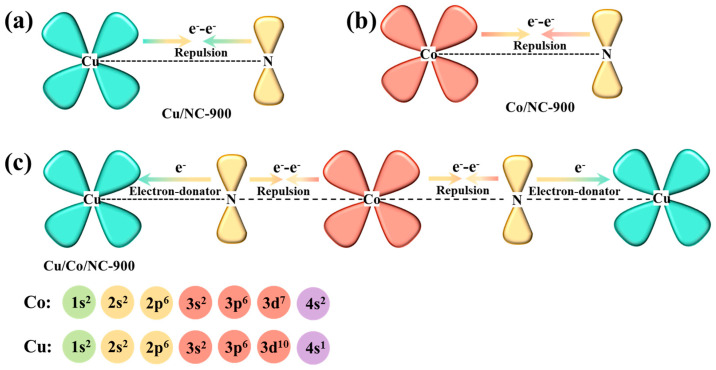
Schematic illustration of the electronic interaction between Cu, Co, and N in (**a**) Cu/NC-900; (**b**) Co/NC-900, and (**c**) Cu/Co/NC-900.

To make an in-depth analysis of the synergistic electronic interactions among Cu, Co, and N in the Cu/Co/NC-900 catalyst, the relevant electron-orbital hybridization diagrams are shown in Figure 4. During the formation of heterostructure systems, the Cu atom usually has a higher d-electron density than the Co atom, and electron transfer occurs at the interface between Co and Cu, resulting in a new electron energy balance [43]. Due to the repulsion between metal atoms and N atoms, the electrons prefer to transfer to the N atoms, resulting in the redistribution and enrichment of electrons on the N atoms in the nitrogen-doped carbon, which enhances the counts of delocalization electrons between Cu/Co and N in Cu/Co/NC-900 [45]. Therefore, the Cu/Co/NC-900 catalyst may exhibit moderate oxygen or hydrogen adsorption energies that can facilitate the electrocatalytic process.

### 2.2. Characterisation of Electrocatalytic HER Properties of Cu/Co/NC Materials

**Figure 5:**
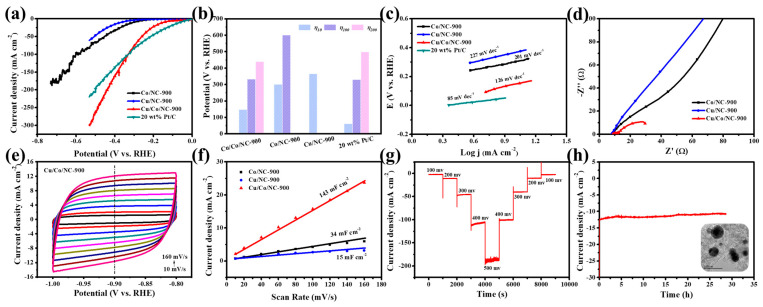
(**a**) HER Polarization curves of Co/NC-900, Cu/NC-900, Cu/Co/NC-900, and 20 wt% Pt/C in alkaline electrolytes; (**b**) The potentials of Cu/Co/NC-900, Co/NC-900, Cu/NC-900, and 20 wt% Pt/C at η_10_, η_100_, and η_200_; (**c**) Tafel plots of Co/NC-900, Cu/NC-900, Cu/Co/NC-900, and 20 wt% Pt/C; (**d**) Nyquist plots of Cu/Co/NC-900, Co/NC-900, and Cu/NC-900 (inset: equivalent circuit diagram); (**e**) CV of Cu/Co/NC-900; (**f**) Electrochemical double-layer capacitance (C_dl_) of Cu/NC-900, Co/NC-900, and Cu/Co/NC-900; (**g**) Multi-step chronoamperometric curves of HER over Cu/Co/NC-900 in alkaline conditions; and (**h**) I-t curve obtained for HER with Cu/Co/NC-900 at an overpotential of 160 mV (Inset: TEM image of Cu/Co/NC-900 sample after 30 h of operation).

Compared to the Co/NC-900 and Cu/NC-900 samples, the Cu/Co/NC-900 sample has a smaller Tafel slope value, revealing that the Cu/Co/NC-900 specimen has the fastest kinetics of electrocatalytic hydrogen reaction. Next, we characterized the charge transfer rate by electrochemical impedance (EIS) for the three samples, Co/NC-900, Cu/NC-900, and Cu/Co/NC-900. Figure 5d shows the Nyquist plot obtained after fitting by the equivalent circuit, revealing that Cu/Co/NC-900 has the smallest R_ct_ relative to Co/NC-900 and Cu/NC-900 and has the fastest rate of electron transfer in the HER reaction. Meanwhile, we investigated the electrochemically active area (ECSA) of the Cu/Co/NC-900 sample, as shown in Figure 5e. We tested the CV of Co/NC-900, Cu/NC-900, and Cu/Co/NC-900 in the non-Faraday region at the sweep speed of 10~160 mV s^−1^ from 1.0 to 0.8 V. Figure 5f shows that the C_dl_ of Cu/Co/NC-900 sample was 143 mF cm^−2^, which is 4.21 and 9.53 times more than that of Co/NC-900 (34 mF cm^−2^), and Cu/NC-900 (15 mF cm^−2^), respectively. The electrochemically active area of the electrocatalytic materials was related to the magnitude of the C_dl_ value, and the positive correlation between the fitting results of C_dl_ and ECSA indicated that the electrochemically active area of the Cu/Co/NC-900 sample was larger, and its exposed active sites were greater during the HER reaction.

Stability is also an important criterion for judging the excellence of a catalyst in real applications. A multi-step timed current test (STEP) was carried out on Cu/Co/NC-900 as shown in Figure 5g, and its current density remained largely stable at different overpotentials, indicating that the catalyst has good stability at both small and large current densities. In addition, we tested the long-term (I-t) stability of the Cu/Co/NC-900 specimen at a constant voltage. Figure 5h intuitively shows that the Cu/Co/NC-900 can work stably in an alkaline electrolyte at a constant voltage of about 12.25 mA cm^−2^ for about 30 h, and the current density is largely stable without degradation. Figure 5h shows the TEM image of the sample after 30 h of operation, in which it is observed that its morphology has not changed, which further indicates the excellent HER electrocatalytic stability of the Cu/Co/NC-900 in an alkaline environment. The electrochemical tests of the samples prepared at different temperatures of thermal treatment are shown in Figure S5, indicating the highest electrocatalytic performance at a temperature of 900 °C. Figure S8 demonstrates the XPS spectra of Cu/Co/NC-900 after the HER stability test. The positions and types of the characteristic peaks appearing in the full spectrum (Figure S8a), C 1s (Figure S8b), N 1s (Figure S8c), Co 2p (Figure S8d), and Cu 2p (Figure S8e) largely remained unchanged. The above results proved that the electrocatalytic performance of the Cu/Co/NC-900 catalyst has not been destroyed after the long-term continuous HER stability test, which reflects the good stability of the catalyst.

### 2.3. Characterisation of Electrocatalytic ORR Properties of Cu/Co/NC Materials

**Figure 6:**
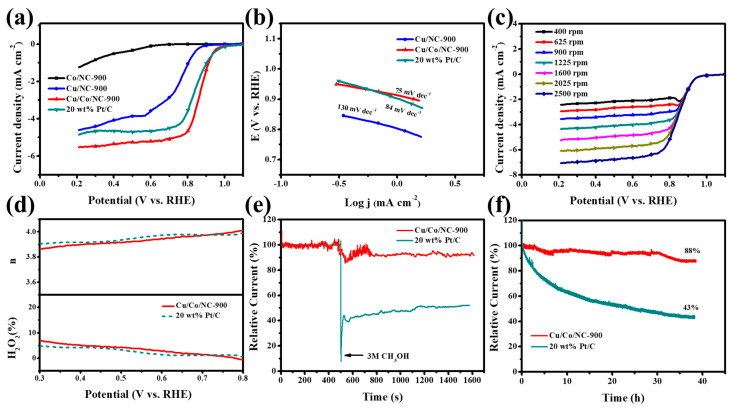
(**a**) LSV curves of ORR with the respective; (**b**) Tafel slope plots for alkaline electrolytes in continuous pass O_2_ at 1600 rpm and sweep rates of 10 mV/s Co/NC-900, Cu/NC-900, Cu/Co/NC-900, and 20 wt% Pt/C; (**c**) LSV curves for Cu/Co/NC-900 catalyst at the scanning rate of 5 mV s^−1^ and speeds of 400–2500 rpm; (**d**) Cu/Co/NC-900 and commercial Pt/C n and H_2_O_2_%; (**e**) Chronoamperometric responses of Cu/Co/NC-900 and 20 wt% Pt/C via injecting 0.3 M methanol 10 mL at 500 s; and (**f**) The I-t chronoamperometric responses of Cu/Co/NC-900 and 20 wt% Pt/C under alkaline conditions.

We found that the Cu/Co/NC-900 material exhibited excellent ORR performance in an alkaline (pH 13) electrolyte saturated with oxygen. Firstly, in the alkaline electrolyte with the continuous passage of oxygen, the LSV curve of ORR of Cu/Co/NC-900 material using RDE was tested and those of Co/NC-900, Cu/NC-900, and 20 wt% Pt/C were also investigated for comparison under the same conditions. As illustrated in Figure 6a, Cu/Co/NC-900 has exceptional ORR activity up to a half-wave potential (E_1/2_) of 0.865 V, which outperforms 20 wt% Pt/C (E_1/2_ = 0.840 V) and Cu/NC-900 (E_1/2_ = 0.745 V), while the Co/NC-900 sample has very poor ORR activity. Figure 6b shows the Tafel slope plot of the as-prepared materials and displays that Cu/Co/NC-900 has a low Tafel slope of only 75 mV dec^−1^, which is smaller than that of 20 wt% Pt/C (84 mV dec^−1^) and Cu/NC-900 (130 mV dec^−1^), demonstrating the fast ORR reaction kinetics of the Cu/Co/NC-900 material. In an attempt to study the reaction mechanism of Cu/Co/NC-900, we measured the LSV at speeds increasing sequentially from 400 rpm to 2500 rpm, as shown in Figure 6c. The K-L plots of Figure S9 derived from Figure 6c, we can intuitively see that there is good parallelism and linearity between ω^−1/2^ and J^−1^, indicating that the n of each oxygen molecule is almost the same at different potentials during the ORR reaction of the Cu/Co/NC-900 material. The n of the Cu/Co/NC-900 material between 0.3 and 0.7 V was calculated to be about 3.87–3.96, which is approaching the theoretical four-electron transfer path for the process of reducing O_2_ to OH^−^, indicating that each oxygen molecule is a four-electron transfer path during the electrocatalytic ORR reaction. Next, we further investigated the Cu/Co/NC-900 material n and H_2_O_2_% using RRDE. From Figure 6d, we can see that its n is in the range of 3.88~3.97, which is close to that of 20 wt% Pt/C, and is compatible with the data from the K-L plot in Figure 6d, which further verifies the four-electron transfer path of its ORR. In addition, the H_2_O_2_% of the Cu/Co/NC-900 material is lower than 7.5% in the test potential realm of 0.3–0.8 V and is equivalent to that of 20 wt% Pt/C, which again confirms that the ORR has a four-electron transfer path. The foregoing conclusions indicate that the Cu/Co/NC-900 material has a high ORR catalytic efficiency.

We characterized the methanol tolerance and stability of the Cu/Co/NC-900 samples. A total of 10 mL of 3.0 M methanol measure was injected into the saturated oxygen alkaline electrolyte at the 500 s mark. It was found that the performance of 20 wt% Pt/C decreased abruptly at the instant of methanol addition, while the current density of the Cu/Co/NC-900 sample showed only a slight decrease (Figure 6e). This shows that the methanol tolerance of the Cu/Co/NC-900 sample was far away from that of 20 wt% Pt/C. Another important criterion to judge the catalyst’s performance in practical applications is the stability of the catalysts, as shown in Figure 6f. The stability tests of Cu/Co/NC-900 and 20 wt% Pt/C were carried out for 40 consecutive hours in an alkaline electrolyte saturated with oxygen (0.1 M KOH). The results showed that the current density of Cu/Co/NC-900 only lost 12%, while that of 20 wt% Pt/C lost 57%, evidencing that the Cu/Co/NC-900 sample has better stability relative to the 20 wt% Pt/C catalyst. The ORR tests of the samples prepared at different heat treatment temperatures are shown in Figure S10, and the optimum half-wave potential (E_1/2_) of Cu/Co/NC-900 is 0.865 V. Finally, we used XPS to characterize the chemical structure of the Cu/Co/NC-900 catalyst after the stability test (Figure S11). It can be seen that the structure of the catalyst was not damaged when the catalyst underwent the continuous stability test up to about 40 h, which further confirmed that the Cu/Co/NC-900 catalyst has good stability. Therefore, the Cu/Co/NC-900 electrocatalyst possesses excellent ORR activity and stability.

## 3. Materials and Methods

### 3.1. Preparation of Materials

#### 3.1.1. Chemicals and Reagents

Copper (II) acetate dihydrate (Cu(CH_3_COO)_2_·H_2_O, A.R.), cobalt(II) acetate tetrahydrate (Co(CH_3_COO)_2_·4H_2_O, A.R.), Ethanol (CH_3_CH_2_OH, A.R.), potassium hydroxide (KOH, A.R.), sodium thiosulfate (Na_2_S_2_O_3_, A.R.), and pyrrole (C_4_H_5_N, C.P), were purchased from Sinopharm Chemical Reagent Co., Ltd., Beijing, China with no further purification. Platinum on activated carbon (20 wt% Pt/C) and Nafion solution (5%) were purchased from Sigma-Aldrich, Shanghai, China. Highly pure water (resistivity > 18 MΩ cm) was provided by a PALL PURELAB Plus system purchased from VEOLIA Water Solutions & Technologies Co., Ltd-ELGA Labwater, Shanghai, China.

#### 3.1.2. Synthesis of Co-CuO_2_/Cu@Ppy

A total of 0.2 g of Cu(CH_3_COO)_2_·H_2_O and 0.25 g of Co(CH_3_COO)_2_·4H_2_O were combined to form solution A after the addition of 40 mL of ultrapure water. Next, 138 μL of C_4_H_5_N was added dropwise to 8.62 mL of ultrapure water to form solution B. Then, solution B was added dropwise to solution A and stirred for 30 min, before being transferred to a polytetrafluoroethylene liner in a hydrothermal reactor. The reaction kettle was lined with PTFE and reacted at 160 °C for 10 h. The powder obtained after the reaction was washed with anhydrous ethanol and ultrapure water, alternately, several times, and then the precursor Co-CuO_2_/Cu@Ppy was dried for 12 h at 60 °C in a vacuum oven. Next, the Co-CuO_2_/Cu@Ppy was immersed for 1 h in a 1 M sodium thiosulfate solution, washed in ultrapure water until neutral, and then dried in a vacuum drying oven at 60 °C for 12 h. The powder was then washed with ultrapure water to produce the precursor Co-CuO_2_@Ppy, which was dried for 12 h in a vacuum drying oven at 60 °C.

#### 3.1.3. Synthesis of Cu/Co/NC

A total of 100 mg of the prepared precursor Co-CuO_2_@Ppy was put into an agate mortar and ground for 10 min and then transferred to a porcelain boat. The precursor sample was then put into a tube furnace and the rate of temperature increase was set to 10 °C/min then held at 900 °C for 2 h in a hydrogen-argon atmosphere before being allowed to cool at the same rate. The black powder eventually obtained was labeled Cu/Co/NC-900.

#### 3.1.4. Synthesis of Cu/NC-900

The Cu/NC-900 specimen was prepared following the same steps as those for Cu/Co/NC-900, with the exception that the raw material Co(CH_3_COO)_2_·4H_2_O was not added.

#### 3.1.5. Synthesis of Co/NC-900

The preparation of Co/NC-900 samples was carried out following the same steps as those for Cu/Co/NC-900, with the exception that the raw material Cu(CH_3_COO)_2_·H_2_O was not added.

### 3.2. Material Characterization

D/MAX-2200PC instrument purchased from Rigaku Corporation, Tokyo, Japan was used to acquire X-ray diffraction (XRD) patterns, using a Cu target. The morphology and microstructure of the materials were observed by field emission scanning electron microscopy (SEM, S4800, Hitachi, Tokyo, Japan) and transmission electron microscopy (TEM, Tecnai G2 F20 S-TWIN, Hillsboro, OR, USA). X-ray photoelectron spectroscopy (XPS) measurements were performed on a Surface Science Instruments spectrometer (XIS SUPRA, Kratos analytical, Manchester, UK). Raman spectra were obtained using a microscope laser confocal Raman spectrometer (Raman, Renishaw-invia, Renishaw, London, UK).

### 3.3. Electrochemical Performance Test Characterization

#### 3.3.1. ORR Electrochemical Characterization

The ORR performance test was completed on the rotating disk electrode (RDE) and rotating ring disk electrode (RRDE) of RIKEN’s MSR electrode rotating device. During the tests, 0.1 M KOH solution (pH 13) was used as the electrolyte, and a platinum counter electrode and a saturated calomel electrode (SCE) were used as the reference electrode and counter electrode, respectively. Prior to the ORR performance test, oxygen was passed for more than 30 min to completely remove the air and obtain a saturated O_2_ solution. The calibration of all potentials requires calibration according to the following formula as $\mathrm{RHE}:\;E_{\mathrm{vs}.\;\mathrm{RHE}}\;=\;E_{\mathrm{vs}.\;\mathrm{SCE}}\;+\;0.2415\;+\;0.059\;\times \;\mathrm{pH}.$ The geometrical area of the working electrode was 0.2376 cm^2^. The RRDE (Rotating Ring Disk Electrode) was tested at 1600 rad/min with scanning rate of 10 mV/s.

#### 3.3.2. HER Electrochemical Characterization

All performance characterizations in HER were acquired at the electrochemical workstation. Tested on a standard three-electrode system, with a saturated calomel electrode (SCE) as reference electrode (RE), a graphite-carbon rod as counter electrode (CE), and a glass-carbon electrode as working electrode (WE). The potentials were all calibrated according to the following formula $E_{\mathrm{vs}.\;\mathrm{RHE}}\;=\;E_{\mathrm{vs}.\;\mathrm{SCE}}\;+\;0.2415\;+\;0.059\;\mathrm{pH}$ and the geometric area of the working electrode was 0.0714 cm^2^.

The relevant electrocatalytic performance tests were carried out in 1 M KOH with continuous nitrogen gas passage. Linear voltammetric scanning (LSV) was carried out with a sweep rate of 5 mV/s and the magnitude of the overpotential required for a current density of 10 mA/cm^2^ to determine the excellence of the catalytic activity of the material. Linear relationship between the logarithm of the current density and the voltage was obtained using the numerical values of the LSV curves according to the equation η = a + b logj Tafel slope plot, and the rate of reaction kinetics of catalytic materials was judged based on the Tafel slope value. Electrochemical active area (ECSA) and double layer capacitance (C_dl_), ECSA were obtained from cyclic voltammetry tests in the non-Faraday current region when the sweep rate was 10~160 mV/s. The larger the ECSA, the more active sites the material has, which indicates a stronger catalytic activity. C_dl_ is obtained from the linear plot obtained in the ECSA, fitting the linear plot. C_dl_ is the half value of the slope of the curve obtained by fitting the linear graph obtained from ECSA. ECSA is proportional to the size of C_dl_, and the size of C_dl_ can be used to express the relationship between the ECSA of the material. The current–time curve (I-t) is the relationship between the current density and the reaction time for hydrogen production under the same voltage, and a current density that does not decay with time indicates that the stability of a material is superior. The multi-step timing curve is the relationship between current density and reaction time for the precipitation of hydrogen, and the current density not decaying with time indicates that the stability of a material is superior. In the multi-step timing current method, the current density is obtained when applying different voltages at different time periods versus the reaction time of hydrogen precipitation, and if the current does not obviously decay at each stage, this indicates that a material also has good stability at different voltages.

## 4. Conclusions

In this work, a novel bifunctional electrocatalyst (Cu/Co/NC) with Cu/Co bimetallic heterostructure was successfully prepared using a combination of hydrothermal route and solid-phase calcination. The synergistic effect of the Cu/Co bimetallic heterostructure effectively modulates the electronic coupling between the two metals and generates a large number of highly efficient M-N_x_ (M = Cu or Co) active sites, which enhances the catalytic activities, while the N-doped carbon carriers enhanced the conductivity and stability of the materials. The resulting Cu/Co/NC material can be employed as an efficient, noble metal-free bifunctional electrocatalyst for HER and ORR, with a low overpotential of 149 mV for HER and a quite high half-wave potential of 0.865 V for ORR at 10 mA/cm^2^ in alkaline electrolyte. This work shown here provides new insights into the development of Cu-/Co-based bifunctional electrocatalysts for the conversion and storage of renewable energy.

## Data Availability

Data are contained within the article and supplementary materials.

## Supplementary Materials

The following supporting information can be downloaded at https://www.mdpi.com/article/10.3390/molecules29132973/s1. Figure S1. XRD patterns of precursor 1 Co-Cu_2_O/Cu@Ppy, precursor 2 Co-Cu_2_O@Ppy, and Cu/Co/NC-900; Figure S2. (a) XRD and (b) Raman patterns of Cu/Co/NC at different heat treatment temperatures; Figure S3. SEM images of catalysts with various heat treatment temperatures (a) 800 °C; (b) 900 °C; and (c) 1000 °C; Figure S4. SEM images of (a) Cu/Co/NC-900; (b) Cu/NC-900; and (c) Co/NC-900; Figure S5. CV of Co/NC-900; Figure S6. CV of Cu/NC-900; Figure S7. Cu/Co/NC samples with different heat treatment temperatures in alkaline conditions: (a) HER polarization curves; (b) Tafel slope; (c) Electrochemical impedance spectroscopy (EIS) Nyquist plots, illustrated with electrical equivalent circuits; and (d) Electrochemical double-layer capacitance (C_dl_) curves; Figure S8. (a) Survey; (b) C 1s; (c) N 1s; (d) Co 2p; and (e) Cu 2p of Cu/Co/NC-900 after stability test; Figure S9. K-L plots corresponding to LSV curves for Cu/Co/NC-900 catalysts at sweep speeds of 5 mV s^−1^ and rotational speeds of 400–2500 rpm; Figure S10. (a) ORR LSV curves and (b) matching Tafel slopes of Cu/Co/NC in alkaline electrolyte with continuous O_2_ pass, 1600 rpm speed, and 10 mV/s sweep rate at different heat treatment temperatures; Figure S11. (a) Survey; (b) C 1s; (c) N 1s; (d) Co 2p; and (e) Cu 2p of Cu/Co/NC-900 after stability test; Table S1. Analytical results of elemental contents in the prepared catalysts determined by XPS; Table S2. Electrocatalytic HER or ORR performance of the Cu/Co@NC-900 electrode compared with Cu-/Co-based electrocatalysts published in recent years in KOH electrolyte. References [46,47,48,49,50,51,52,53,54,55,56,57] are cited in the supplementary material.
