# Supplementary material for: Electronic-Structure-Modulated Cu,Co-Coanchored N-Doped Nanocarbon as a Difunctional Electrocatalyst for Hydrogen Evolution and Oxygen Reduction Reactions
Source: Molecules. 2024 Jun 22;29(13):2973. doi: 10.3390/molecules29132973 (PMC11243191; doi:10.3390/molecules29132973)
Supplement: Supplementary file 1 [file molecules-29-02973-s001.zip › molecules-3059590-supplementary.pdf]

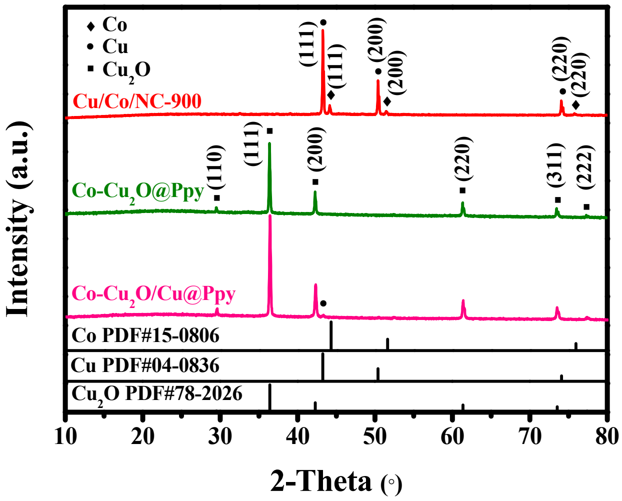

Figure S1. XRD patterns of precursor 1 Co-Cu2O/Cu@Ppy, precursor 2 Co-Cu2O@Ppy and Cu/Co/NC-900.

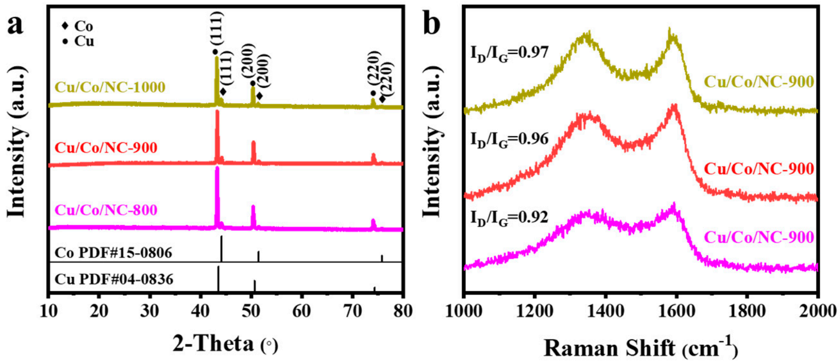

Figure S2. (a) XRD and (b) Raman patterns of Cu/Co/NC at different heat treatment temperatures.

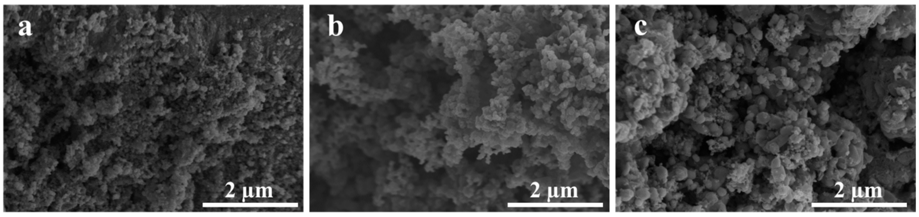

Figure S3. SEM images of catalysts with various heat treatment temperatures (a) 800 °C; (b) 900 °C; (c) 1000 °C.

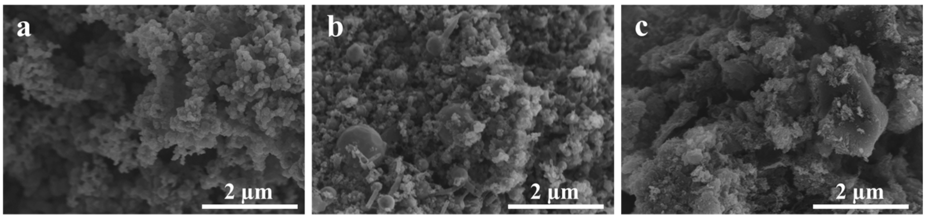

Figure S4. SEM images of (a) Cu/Co/NC-900; (b) Cu/NC-900; (c) Co/NC-900.

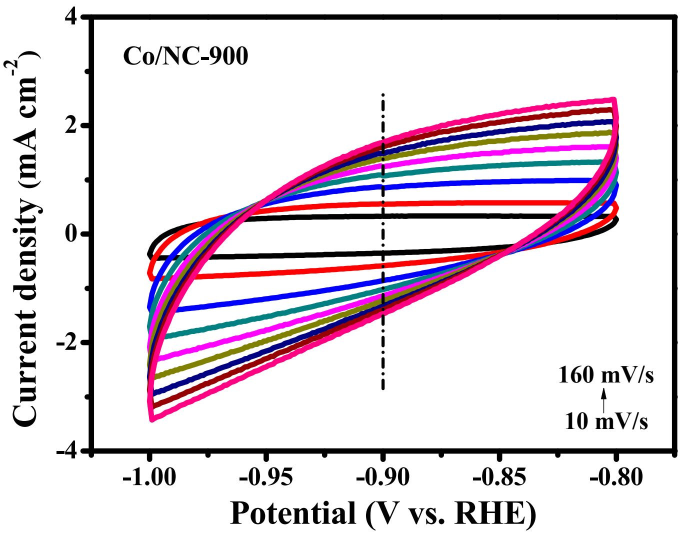

Figure S5. CV of Co/NC-900.

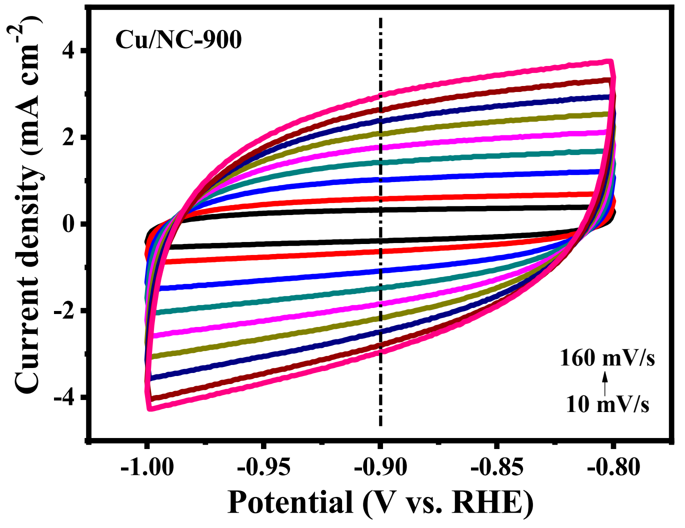

Figure S6. CV of Cu/NC-900.

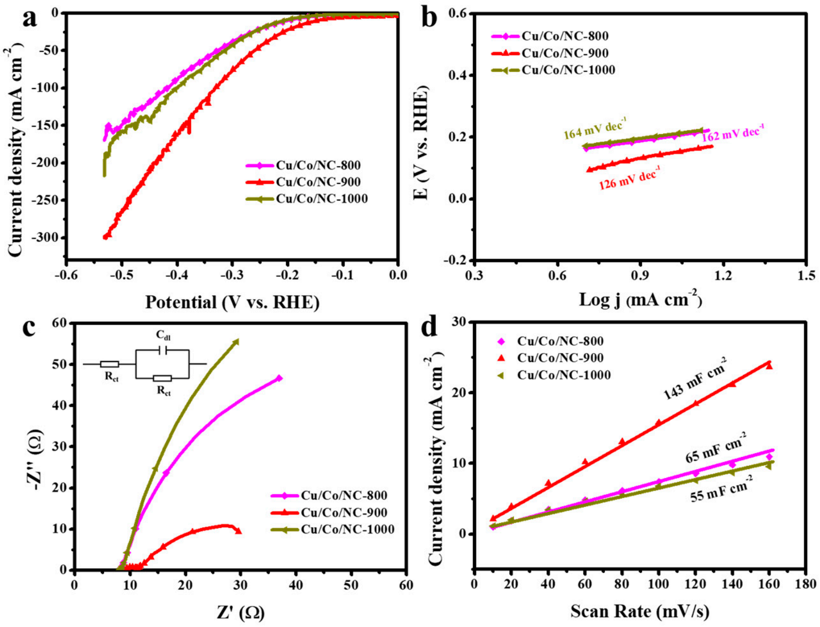

Figure S7. Cu/Co/NC samples with different heat treatment temperatures in alkaline conditions: (a) HER polarization curves; (b) Tafel slope; (c) Electrochemical impedance spectroscopy (EIS) Nyquist plots, illustrated with electrical equivalent circuits; (d) Electrochemical double-layer capacitance (C_{dl}) curves.

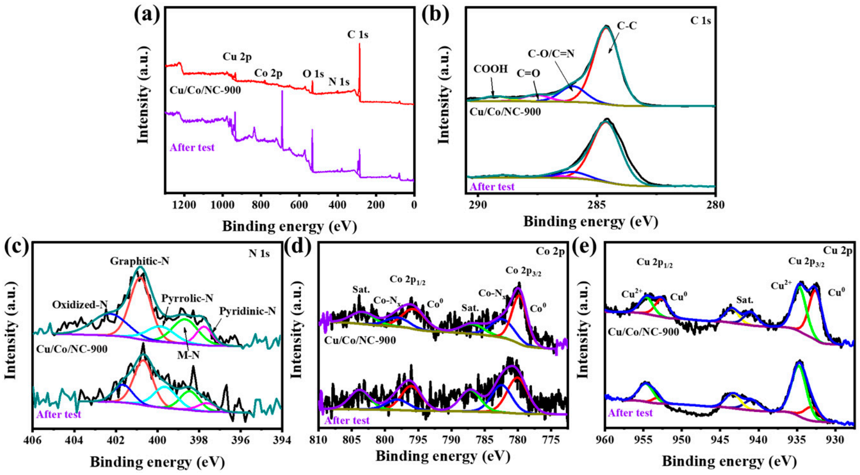

Figure S8. (a) Survey; (b) C 1s; (c) N 1s; (d) Co 2p; (e) Cu 2p of Cu/Co/NC-900 before and after stability test.

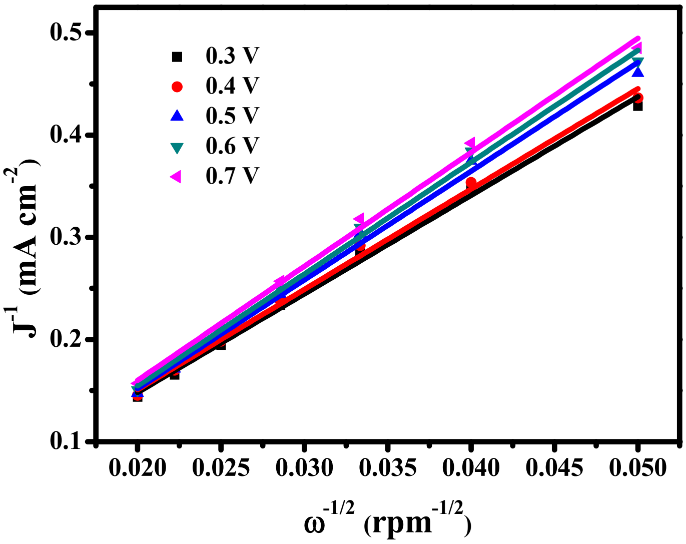

Figure S9. K-L plots corresponding to LSV curves for Cu/Co/NC-900 catalysts at sweep speeds of 5 mV s^{-1} and rotational speeds of 400-2500 rpm.

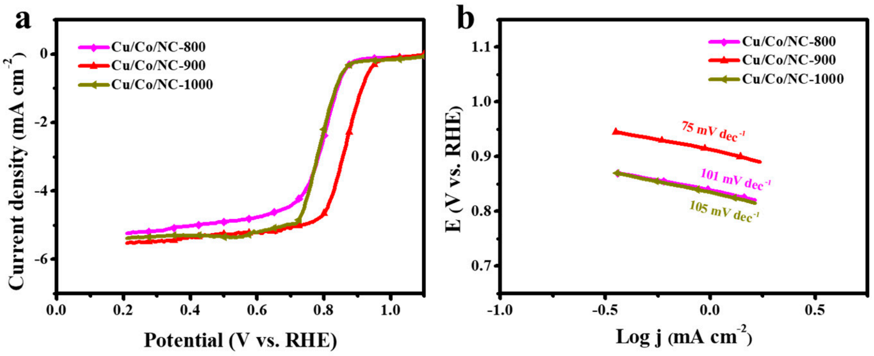

Figure S10. (a) ORR LSV curves and (b) matching Tafel slopes of Cu/Co/NC in alkaline electrolyte with continuous O_2 pass, 1600 rpm speed and 10 mV/s sweep rate at different heat treatment temperatures.

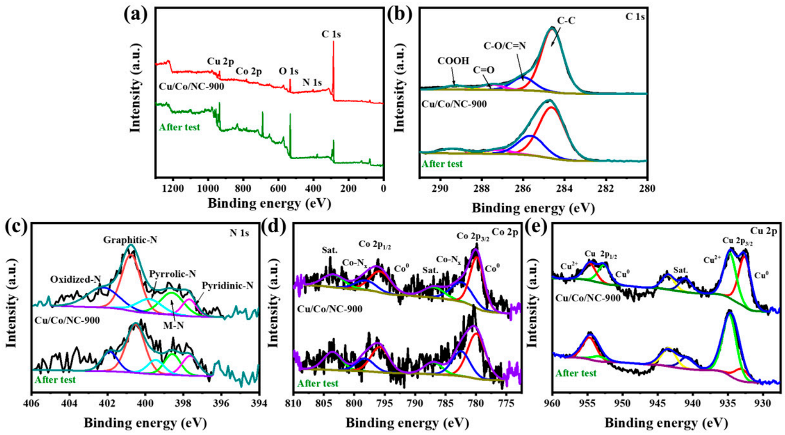

Figure S11. (a) Survey; (b) C 1s; (c) N 1s; (d) Co 2p; (e) Cu 2p of Cu/Co/NC-900 before and after stability test.

Table S1. Analytical results of elemental contents in the prepared catalysts determined by XPS.

Samples	Composition (at %)	
	Cu	Co
Cu/Co/NC-900	**1.01**	**0.40**
Cu/NC-900	**0.14**	**-**
Co/NC-900	**-**	**4.45**
After HER test Cu/Co/NC-900	**3.17**	**0.24**
After ORR test Cu/Co/NC-900	**3.48**	**0.47**

Table S2. Electrocatalytic HER or ORR performance of the Cu/Co@NC-900 electrode compared with Cu-/Co-based electrocatalysts published in recent years in KOH electrolyte.

	Catalysts	HER	ORR	References
		η_{10} (mV vs RHE)	$E_{1/2}$ (V vs RHE)	
1	*Cu/Co/NC-900*	*149*	*0.865*	*In this work*
2	CuCoS/N-rGO-50	86	-	Int. J. Hydrog. 2024, 65: 704-716.
3	CoP/Co-N-C	190	0.89	Chem. Eng. J. 2024: 152301.
4	Cu,Co/NSC	159	0.95	Adv. Funct. Mater. 2024, 34(10): 2311664.
5	SC-Cu_{SA}-NC	124	0.83	Compo Part B-Eng, 2023, 253: 110575.
6	Cu-NPC-KOH	-	0.83	Int J Hydrogen Energ. 2024, 69: 21-30.
7	$Cu_{81}(Ni,Co)_{19}$	35	-	J Alloys Compounds 2024, 995: 174790.
8	BN/Cu/CNT	-	0.78	Int J Hydrogen Energ. 2023, 48(53): 20368-20377.
9	N-MoO/Cu	40	-	Int J Hydrogen Energ. 2024, 66: 103-109.
10	MoS_2/Cu-SAs	114	-	Small Struct, 2023, 4(8): 2300010.

11	Cu CP	594	-	Dalton T.2023, 52(26): 8850-8856
12	Cu-ZrO_{3-x}@ N-BPCNFs	-	0.856	Small, 2023, 19(15): 2206823.
13	SA-CoCu@Cu/CoNP	-	0.88	Adv. Energy Mater. 2021, 11(17): 2100303.
